# Reducing agents facilitate membrane patch seal integrity and longevity

**DOI:** 10.1080/19336950.2023.2297621

**Published:** 2023-12-28

**Authors:** Damayantee Das, Anson Wong, Timothy N. Friedman, Bradley J Kerr, Harley T. Kurata, Shawn M. Lamothe

**Affiliations:** a Dept. of Pharmacology, Alberta Diabetes Institute, University of Alberta, Edmonton, Canada; b Neuroscience and Mental Health Institute, University of Alberta, Edmonton, Canada; c Department of Anesthesiology and Pain Medicine, University of Alberta, Edmonton, Canada

**Keywords:** patch clamp, reducing agents, DTT, TCEP, giga-ohm, quality, seals, integrity, longevity

## Abstract

The patch clamp method is a widely applied electrophysiological technique used to understand ion channel activity and cellular excitation. The formation of a high resistance giga-ohm seal is required to obtain high-quality recordings but can be challenging due to variables including operator experience and cell preparation. Therefore, the identification of methods to promote the formation and longevity of giga-ohm seals may be beneficial. In this report, we describe our observation that the application of reducing agents (DTT and TCEP) to the external bath solution during whole-cell patch clamp recordings of heterologous cells (HEK and LM) and cultured primary cells (DRG neurons) enhanced the success of giga-ohm seal formation. Reducing agents also maintained the integrity of the seal for longer periods of time at strong hyperpolarizing voltages, whereas an oxidizing agent (H_2_O_2_) appeared to have the opposite effect. In summary, we report a useful tool to improve the quality of patch clamp recordings that may be helpful in certain experimental contexts.

## Introduction

The patch clamp technique was developed in the late 1970s to early 1980s and revolutionized the understanding of ion channels and electrical activity of excitable cells [1–3]. Technical and physical complexities of patch clamping present challenges and extensive training is needed to become proficient with the technique. A common challenge for novice patch clampers is the need to form and maintain high resistance “giga-ohm” seals between the cell membrane and the patch pipette [4]. While learning the technique, consistently obtaining giga-ohm seals is often a limiting factor in generating data, and instability of seals can limit the duration and quality of recordings.

In addition to user knowledge, technique, and experience, the success of a giga-ohm seal may depend on other factors including cell quality and preparation, materials, and solutions used for recording, and even skilled electrophysiologists can be challenged by experimental days when cells do not form optimal seals. Variables commonly considered include the type of glass (i.e. borosilicate, flint, and soda lime), and fire polishing of electrode tips [5]. Ionic composition of external and internal solutions may also significantly affect seal formation. It is generally accepted that millimolar concentrations of Mg^2+^ and Ca^2+^ in the recording solutions promote seals, where Ca^2+^ has been proposed to enhance salt bridge formation between polar residues on the membrane and glass of the patch pipette [3,6]. However, other groups report strong seal formation in the presence of the powerful divalent chelator EDTA, suggesting that this is not an absolute requirement. It has also been demonstrated that 100 mM K^+^ in the pipette solution negatively affected membrane seals, whereas the absence of K^+^ was beneficial for seal formation [7]. Hydrogen (H^+^) ions also influence membrane-patch interaction [6], where lower pH values (4.5) in pipette solutions were reported to inhibit seal formation compared to pH values ranging from 6.0 to 7.8 in protoplast preparations [7].

Although manipulation of ions or conditions in patch clamp solutions is a straightforward task, isolation of various ionic currents often requires the inclusion/exclusion of specific ions and pH ranges, which may compromise the formation and integrity of a sustained giga-ohm seal. This highlights the benefit of identifying other factors that can enhance seal formation for patch clamp recordings. In this study, we report our observation that reducing agents in the extracellular bath solution during patch clamp recordings enhances seal formation and longevity.

## Methods and materials

### Molecular and cellular biology

Mouse LM(tk-) fibroblast cells (ATCC CCL-1.3, referred to as LM cells throughout), Human embryonic kidney (HEK) −293 cells and dissociated mouse DRG neurons were used for electrophysiological recordings. LM and HEK cultured cells were maintained in an incubator at 37.0°C and 5% CO_2_ in DMEM supplemented with 10% FBS and 1% penicillin/streptomycin. Cells were passaged at 70%–80% confluence into 12 or 24 well plates, then transfected with 600 ng of the voltage-gated potassium channel, Kv1.5 (*Homo Sapiens*; accession: NM 012970.3) and 200 ng of the fluorescent protein, GFP (Addgene: 54705), to identify transfected cells for electrophysiological recordings. As per the manufacturer’s instructions, cells were transfected with 2 μl Jetprime (Polyplus) transfection reagent per well (12 or 24 well plates) with 50 μl Jetprime buffer and the respective plasmids (Kv1.5, GFP) for 6 h. Following transfections, cells were seeded onto 22X22–1.5 coverslips (Fisher Scientific 12541B) in six well plates 24 h before experiments were performed.

### Tissue harvesting and dissociated DRG neuron cultures for electrophysiological recordings

Dorsal Root Ganglion Neurons (DRGNs) were acquired from female mice, using a modified protocol from previously described work [8]. Briefly, the animals were euthanized by Euthanyl® (sodium pentobarbital) injected intraperitoneally. After injection, animals were monitored for levels of consciousness and dissections did not proceed until no response to toe pinch or corneal contact was observed. Cardiac punctures were performed to confirm euthanization, and animals were perfused with 10 mL of ice-cold saline. Perfused animals underwent spinal laminectomies and gross dissection of the spinal cord to expose the DRGs. DRGs were micro-dissected from the spinal column, taking care to remove as much residual nerve as possible while avoiding damage to the DRG. Isolated DRGs were placed in ice-cold HBSS -/- (Gibco 14,175–095) until dissections were completed. To acquire single-cell suspensions of DRGN, the HBSS -/- was replaced with a warmed dilution of STEMxyme® I (Worthington, LS004106) and DNase (Worthington, LS002007) in HBSS -/- and incubated in a 37°C water bath for approximately 45 min. Following digestion, enzyme activity was quenched with equal volumes of low ovomucoid (Worthington, LS003086) and mechanically titrated with a P1000 pipette until tissue was fully dissociated. The cell suspension was filtered through a 70 μm mesh filter (Biologix, 15–1070) and gently layered on top of a 20% Bovine Serum Albumin (BSA) solution (Sigma, A4161). This layered gradient was spun at 300 × G for 10 min at room temperature to pellet neuronal cells and remove cellular debris (mainly myelin). Debris was gently removed, and the cell pellet was resuspended in a small volume of 0.5% BSA in HBSS -/- and quantified for neuronal yield with a hemocytometer. The cell suspension was adjusted to 1000 cells/100 μL, and 100 μL of this suspension was added to equilibrated media in a 24 well (Falcon 353,047), poly-D-lysine coated plate (Sigma, P6407). For electrophysiological recordings, cells were plated onto glass coverslips (Fisher 1,254,583) identically coated in poly-D-lysine and transferred into the electrophysiological recording setup at experimental timepoints. Unless otherwise stated, plated neurons were incubated at 37°C with 5% CO_2_ for 48 h.

### Electrophysiological recordings

Patch clamp pipettes were manufactured from soda-lime glass capillaries (Fisher Scientific), using a Sutter *P*-97 puller (Sutter Instrument). Patch-pipette tips were not fire-polished. When filled with internal recording solutions, patch pipettes had a tip resistance between 1 and 3 MΩ. Recordings were filtered at 5 kHz, sampled at 10 kHz, with manual capacitance compensation and 80% series resistance compensation. Current traces were obtained with an Axopatch-200B amplifier, Digidata 1440A digitizer and recordings were processed using Clampex 10.7 software (Molecular Devices). The external (bath) solution had the following composition: 135 mM NaCl, 5 mM KCl, 1 mM CaCl_2_, 1 mM MgCl_2_, 10 mM HEPES, and was adjusted to pH 7.3 with NaOH. The internal (pipette) solution had the following composition: 135 mM KCl, 5 mM K-EGTA, 10 mM HEPES and was adjusted to pH 7.2 using KOH. Images of Dorsal Root Ganglion neurons in the recording chamber were acquired using a high-resolution USB2.0 CMOS Camera (1280 × 1024, Thorlabs, DCC1645C) and ThorCam™ software.

### Drug solutions

Dithiothreitol (DTT) was obtained from Fisher Scientific (BP172–25), prepared as a 200 mM stock solution in ddH_2_O and diluted in recording solutions to appropriate concentrations on the day of experiments. Tris-(2-Carboxyethyl)phosphine hydrochloride (TCEP-HCL) was obtained from Thermo Fisher Scientific (Product #20490), prepared as a 50 mM stock in ddH_2_O solution and adjusted to pH 7.2 with NaOH. The stock solution was diluted in external recording solution to desired concentrations on the day of experiments. Hydrogen Peroxide (H_2_O_2_) was obtained from Thermo Fisher Scientific (CAS 7722-84-1), prepared as a 50 mM stock in H_2_O and diluted in extracellular recording solutions to appropriate concentrations on the day of experiments.

### Statistical analyses

Data from all experimenters were grouped categorically into seal resistances above or below 0.5 gigaOhms. Seal success was compared against ambient vs. reducing (DTT or TCEP) and ambient vs. oxidized (H_2_O_2_) patch clamping conditions and the Chi-squared/Fisher’s exact tests, using SPSS statistics 29.0.1.0, evaluated the significance between the categorical groups. All tests were two-sided and a p-value of < 0.05 was considered significant.

## Results

### Reducing agents promote seal formation of heterologous cells

**Figure 1.**
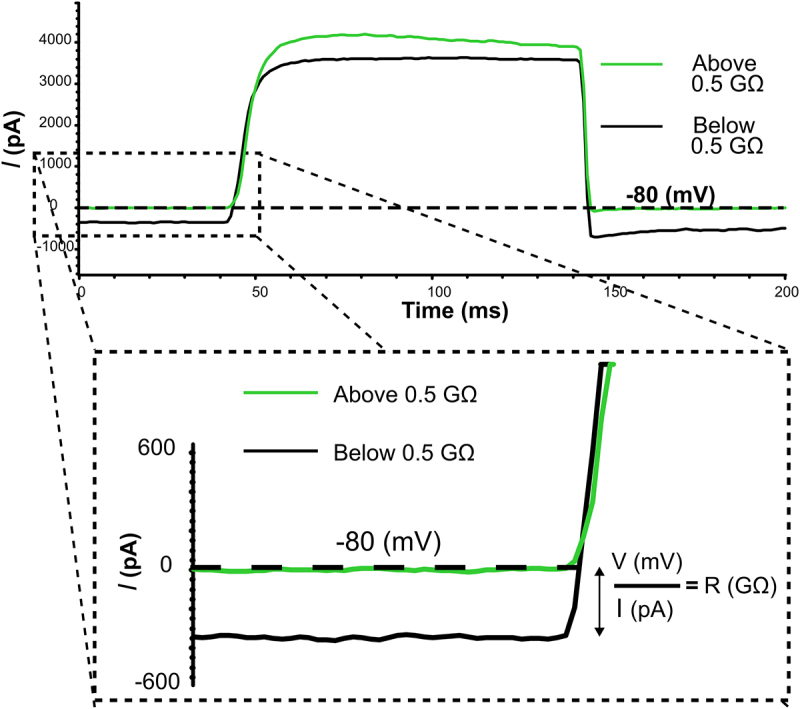
Currents and schematic representation of seal resistance calculation. the current (pA) vs. time (ms) graph shows representative current traces from two-independent cells upon depolarization to 20 mV from a − 80-mV holding voltage immediately after whole-cell break-in. Exemplar traces from cells that maintain a seal resistance above 0.5 GΩ (no current leak) are depicted in green, while cells that have a seal resistance below 0.5 GΩ (current leak) are depicted in black. The inset below illustrates seal resistance (GΩ) calculation based on holding voltage (mV) divided by leak current (pA) in accordance with Ohm’s law.

**Figure 2.**
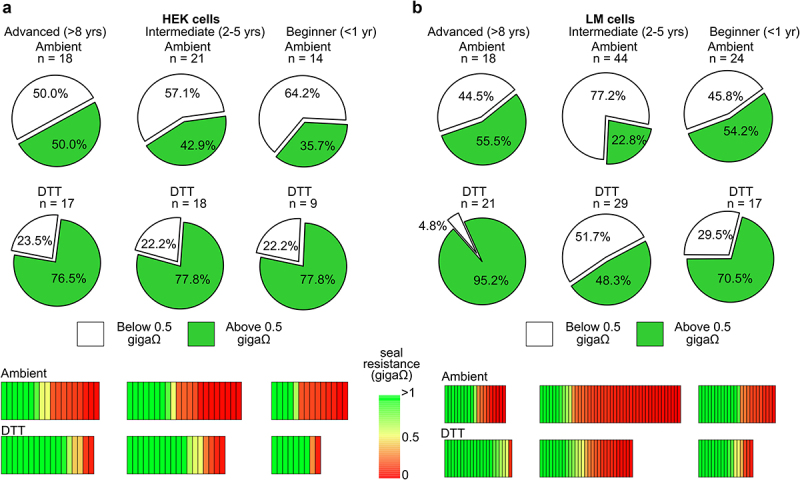
DTT enhances seal formation in HEK and LM cells. pie graphs indicating the percentage of seal resistances “above 0.5 GΩ” (in green) or “below 0.5 GΩ” (in white) from HEK (a) or LM (b) cells recorded, in control (ambient) or reducing (200 μM DTT) conditions. Data was acquired from three independent experimenters and categorized based on years of patch clamp experience from each operator (advanced, >8 years; intermediate, 2–5 years; beginner, <1 year). Number of cells recorded from each experimenter and condition is reported above the respective pie graph. Percent seal improvements in the presence of DTT compared to ambient conditions from each experimenter are reported below the respective pie graph. Heat maps under the respective experimenter illustrate the seal resistance of each individual cell recorded in either ambient or reducing (200 μM DTT) conditions.

**Figure 3.**
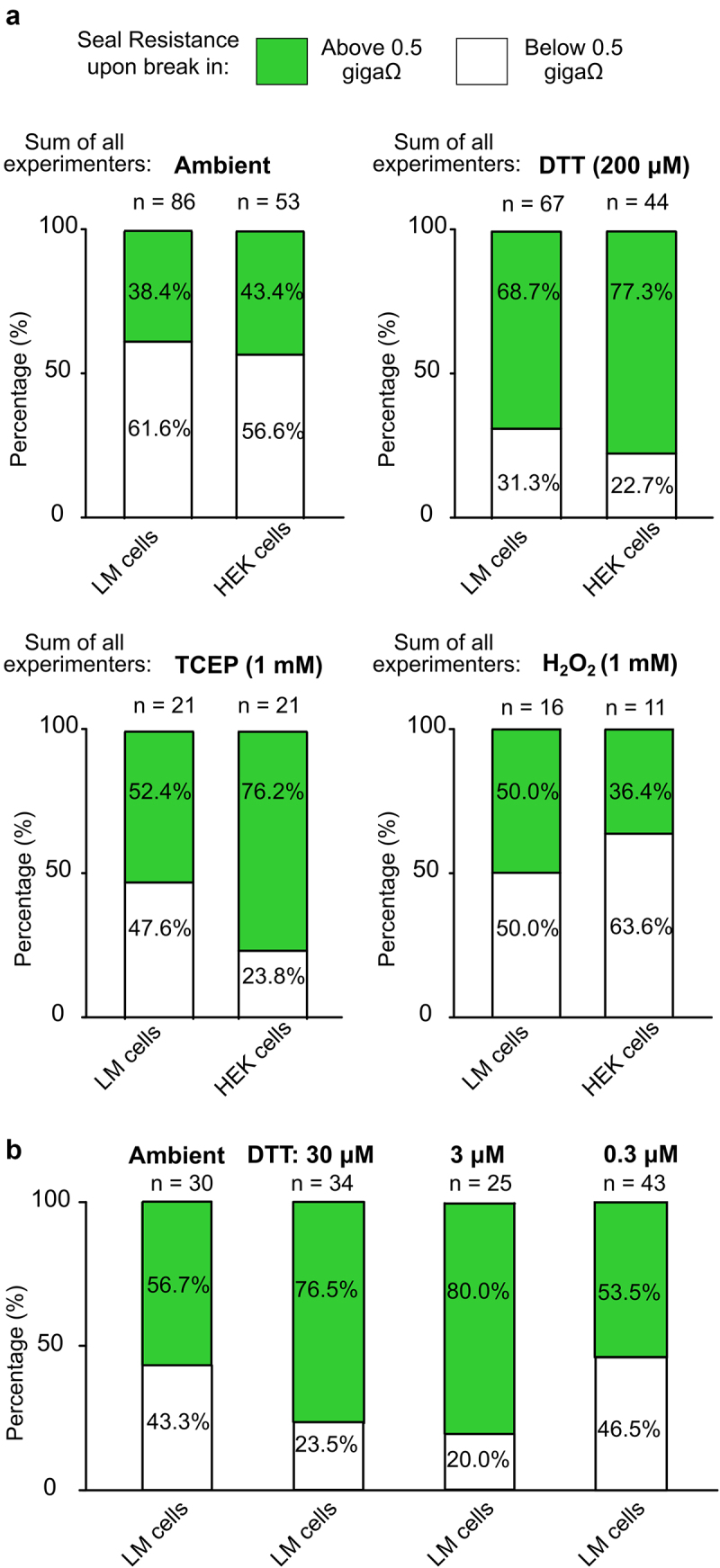
Extracellular redox conditions affect the integrity of seals in heterologous cells. a) bar graphs of summarized data from all experimenters depicting the percentage of seal resistances “above 0.5 GΩ” (in green) or “below 0.5 GΩ” (in white) after break-in from HEK or LM cells recorded in control (ambient), reducing (200 μM DTT or 1 mM TCEP) or oxidizing (1 mM H_2_O_2_) conditions. Number of recorded HEK or LM cells from each condition is reported above the respective bar. b) bar graphs depicting the percentage of LM cells with calculated seal resistances “above 0.5 GΩ” (in green) or “below 0.5 GΩ” (in white) recorded in control (ambient) conditions or in the presence of varying concentrations (30 µM, 3 µM and 0.3 µM) of DTT. Number of recorded LM cells for each condition is reported above the respective bar.

Previous investigations into redox modulation of Kv channels led to anecdotal observations that reducing agents markedly improved outcomes in patch clamp experiments, and we sought to formalize this observation to be useful for other electrophysiologists [9]. Patch clamp recordings were performed by three different lab members with varying degrees of patch clamp experience, each operating a similarly equipped patch clamp rig. Cells were recorded with a voltage step protocol in ambient redox conditions or in the presence of DTT, TCEP (reducing agents) or H_2_O_2_ (oxidizing agent) prepared and applied to the external bath solution prior to recording by the experimenter. Experimenters sought to obtain giga-ohm seals followed by whole-cell break-in and current recordings. K^+^ currents from Kv1.5 channels were used to assess the quality of the seal because we had previously observed that application of reducing agents does not affect macroscopic Kv1.5 current [9]. The quality of the seal at the beginning of each recording was arbitrarily clustered into seal resistances above or below 0.5 giga-ohms based on the holding current at −80 mV (Figure 1) and categorized based on experience of the operator (advanced, >8 years; intermediate, 2–5 years; or beginner, <1 year, Figure 2). In ambient conditions, the advanced, intermediate, and beginner experimenters obtained seal resistances above 0.5 giga-ohms with 55.5%, 22.8%, and 54.2% of recordings from LM cells (Figure 2b) and 50.0%, 42.9%, and 35.7% from HEK cells (Figure 2a), respectively. In recordings made with 200 μM DTT in the bath solution, there was a marked improvement in seal resistance after break-in for all experimenters. As illustrated in Figure 2, in the presence of DTT the advanced experimenter saw a 39.7% improvement, the intermediate experimenter saw a 25.5% improvement and the beginner saw a 16.3% improvement in seal formation compared to ambient control conditions while recording from LM cells. A similar outcome was obtained while recording from HEK cells where the advanced, intermediate, and beginner experimenters reported 26.5%, 34.9%, and 42.1% enhancements in DTT, respectively. Overall, when combining the results of all experimenters, there was a statistically significant improvement in seal quality when DTT is present in the bath solution compared to control ambient conditions in HEK and LM cells (Figure 3a) (Table 1).

**Table 1.** Success of seal formation from all experimenters recorded in ambient vs. reducing (DTT and TCEP) or ambient vs. oxidizing (H_2_O_2_) conditions from different cell types. Observed values, (probability %), [chi-squared] p-values, and {Fisher’s exact^b^} p-values are reported. Statistically significant p-values (<0.05) are bolded.

	HEK cells			LM cells			DRG neurons		
Conditions	Above 0.5 GΩ	Below 0.5 GΩ	p-value [χ2] {^b^}	Above 0.5 GΩ	Below 0.5 GΩ	p-value [χ2]	Above 0.5 GΩ	Below 0.5 GΩ	p-value [χ2] {^b^}
**Ambient**	23.00 (43.40)	30.00 (56.60)		33.00 (38.40)	53.00 (61.60)		36.00 (45.00)	39.00 (55.00)	
*Reducing*									
**DTT**	34.00 (77.30)	10.00 (22.70)	**<0.01** [11.39]	46.00 (68.70)	21.00 (31.30)	**<0.01** [13.83]	37.00 (66.50)	17.00 (33.50)	**0.020** [5.381]
**TCEP**	16.00 (76.20)	5.00 (23.80)	**0.011** [6.489]	11.00 (52.40)	10.00 (47.60)	0.242 [1.37]	17.00 (81.00)	4.00 (19.00)	**0.007** [7.204] **{0.012^b^}**
*Oxidizing*									
**H**_**2**_**O**_**2**_	4.00 (36.4)	7.00 (63.6)	0.667 [0.185] {0.748^b^}	8.00 (50.00)	8.00 (50.00)	0.384 [0.759]			

To determine whether the enhanced seal formation was due to the reducing activity of DTT, we performed additional recordings in the presence of 1 mM TCEP (a membrane impermeable reducing agent) or 1 mM H_2_O_2_ (an oxidizing agent). TCEP in the bath led to a statistically significant improvement in seal formation in HEK cells, while the oxidizing agent, H_2_O_2_, did not promote the formation of seals in LM or HEK cells compared to ambient control conditions (Figure 3a) (Table 1).

We tested a range of DTT concentrations in the bath solution to determine a saturating and optimal concentration for the enhanced seal forming effect in reducing conditions. Compared to ambient (control) conditions, the success of obtaining seals above 0.5 giga-ohms in LM cells was greatly improved with 30 μM and 3 μM DTT but not 0.3 μM DTT, indicating that the effectiveness of the reducing conditions requires only modest concentrations of DTT (Figure 3b).

### Reducing agents sustain seal integrity at strong hyperpolarizing voltages

**Figure 4.**
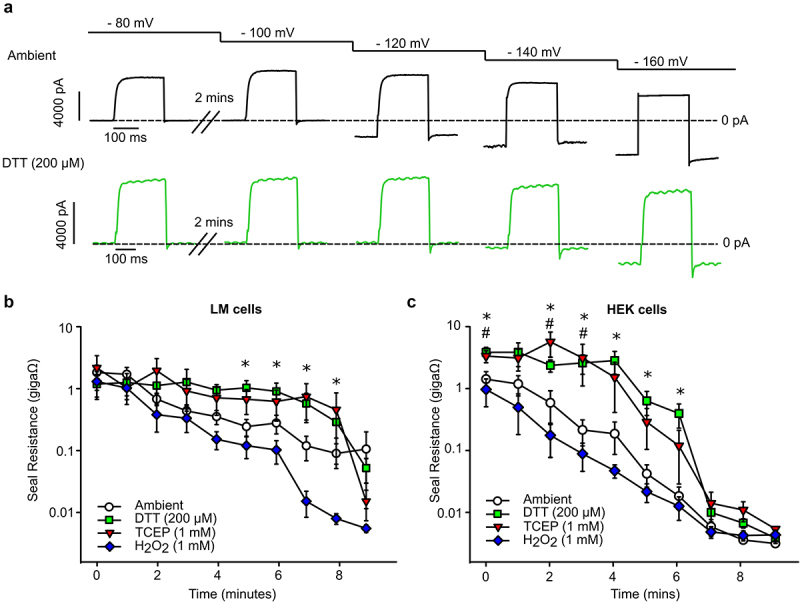
Extracellular redox conditions affect the longevity of seals in heterologous cells. a) Representative current traces from cells recorded in either control (ambient, black) or reducing conditions (200 μM DTT, green) using a sequence of stepwise holding voltages ranging from −80 mV to −160 mV, where each voltage was held for 2 minutes. Cells were pulsed every minute to 20 mV for 100 ms from the respective holding voltage to calculate the seal resistance. Dotted lines denote zero current level. (b,c) time course (mins) of seal resistance decay (giga Ω) from LM (b) or HEK (c) cells in the indicated conditions. Voltage steps at 2-minute intervals are illustrated below the representative current traces and above the graphs as reference. A kruskal-Wallis ANOVA on ranks between all groups with a multiple comparisons test versus ambient control conditions (Dunn’s method) were performed to evaluate statistical significance at each time point. P-value (<0.05) from Dunn’s multiple comparisons tests were reported above the respective time point on each graph, (* = DTT, ^#^ = TCEP).

Seal breakdown is an additional technical challenge that arises with prolonged recording of cells and can be accelerated by voltage clamping at supraphysiological voltages common in biophysical studies of ion channels. To investigate whether reducing agents preserve integrity of seals for longer periods of time and at more extreme experimental conditions, we recorded cells using a sequence of holding voltages ranging from −80 mV to −160 mV, with each negative voltage held for 2 min. Cells were pulsed every minute to 20 mV for 100 ms from the respective holding voltage, and the seal resistance was calculated (Figure 4a). In control (ambient) conditions there was a progressive decline in seal resistance from both LM and HEK cells as the holding voltage became more negative. In the presence of reducing agents DTT (200 μM) and TCEP (1 mM), the seal resistance in LM and HEK cells declined at a slower rate compared to ambient conditions such that a significant decline in seal quality was not observed until cells were held at strong negative voltages (−120 to −160 mV in LM cells and −100 to −140 mV in HEK cells) for more than 2 min (Figure 4b,c). These results indicate that the quality of membrane-patch seals is preserved in extracellular reducing conditions.

### Reducing agents promote seal formation of mouse primary dorsal root ganglion neurons

**Figure 5.**
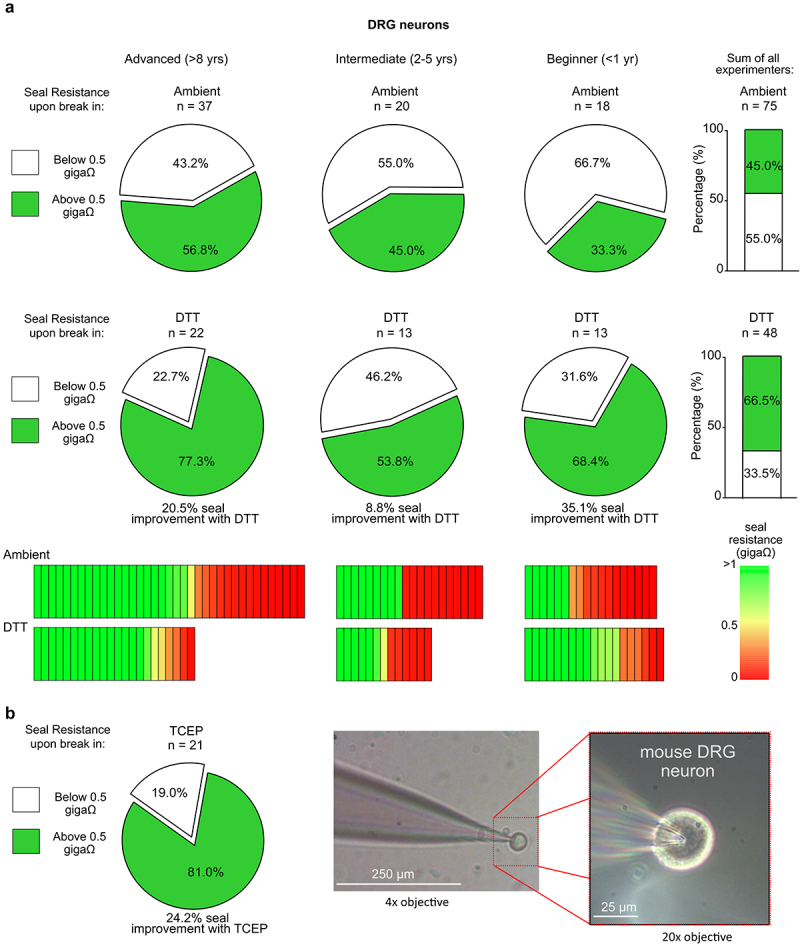
Extracellular reducing agents enhance seal formation in mouse dorsal root ganglion (DRG) neurons. (a) pie graphs representing the percentage of seal resistances “above 0.5 GΩ” (in green) or “below 0.5 GΩ” (in white) immediately after whole-cell break-in from dorsal root ganglion (DRG) neurons recorded in control (ambient) or reducing (200 μM DTT) conditions. Data was acquired from three independent experimenters and categorized as described in figure 2. Bar graphs (*right* of pie graphs) are summarized data from all experimenters in control (ambient) conditions or in the presence of 200 µM DTT. Heat maps under the respective experimenter illustrate the seal resistance of each individual cell recorded in either ambient or reducing (200 μM DTT) conditions. Number of recorded DRG neurons from each operator and condition is reported above the respective pie or bar graph. (b) Seal success in TCEP (1 mM) is presented for the advanced experimenter, along with an exemplar bright-field microscopic image with 4× objective (magnified with 20× objective) demonstrating a giga-ohm seal formed between the glass pipette tip and the DRG neuron.

Formation of giga-ohm seals is also often difficult after enzymatic dissociation to isolate primary cells. To determine whether reducing agents can contribute to successful acquisition of giga-ohm seals in primary cell cultures, recordings from isolated mouse DRG neurons with soma diameters ranging from small (< ~25 um) to large (> ~40um) were similarly generated by three independent experimenters, using cells from the same preparations. Consistent with observations in heterologous (LM and HEK) cells, DTT promoted the formation of giga-ohm seals in DRG neurons. As illustrated in Figure 5, while the advanced patch clamper obtained seals above 0.5 giga-ohms 56.8% of the time in ambient control conditions, there was a marked improvement in seal formation in the presence of both 200 μM DTT (77.3% above 0.5 giga-ohms, Figure 5a) and 1 mM TCEP (81.0% above 0.5 giga-ohms, Figure 5b). Improvement was also seen for the other patch clampers such that an improvement from 45.0% to 53.8% of seals above 0.5 giga-ohms from the intermediate experimenter, and 33.3% to 68.4% from the beginner experimenter were obtained when DRG neurons were recorded in the presence of 200 μM DTT. As a sum from all experimenters recording in ambient control conditions, seals above 0.5 giga-ohms were obtained approximately half of the time (45%). Conversely, with DTT in the bath solution, experimenters obtained seals above 0.5 giga-ohms approximately two-thirds of the time (66.5%) (Figure 5a). Overall, extracellular application of reducing agents, DTT and TCEP, significantly improved the formation of patch clamp seals for all experimenters recording from DRG neurons (Figure 5a) (Table 1).

## Discussion

Success with patch clamp experiments requires the formation of high-quality giga-ohm resistance seals between the patch pipette and the cell membrane. Giga-ohm seals are important in all patch clamp configurations and can affect outcomes including the fidelity and speed of voltage clamp. Seal quality also influences electrical noise, which may be especially important in experiments such as single channel recordings where a weak seal with substantial leak may produce significant noise that obscures single channel currents [10]. High resistance seals are required to accurately measure the electrical behavior of excitable cells; tighter seals lead to more faithful experimental control and measurements of membrane potential and currents [11,12]. Also, the efficiency of high-throughput electrophysiological assays (planar patch clamp chips) for use in drug development and discovery are contingent upon the generation of high-resistance seals as data is discarded if the membrane seal resistance is below a certain threshold [13]. More generally, every experienced patch clamper has experienced struggles of cell preparations that are reluctant to form seals, become fragile upon break-in, or break down before a challenging voltage step protocol can be completed. Where compatible with experimental objectives, we hope that reporting this unexpected effect of DTT or other reducing agents will provide an additional useful tool to overcome experimental challenges that may be encountered while patch clamping. These examples highlight the practicality of finding new methods to assist patch clampers with forming consistent giga-ohm seals during experimentation.

We recently reported that extracellular reducing conditions strongly influence gating of the homomeric voltage-gated potassium channel, Kv1.2, compared to other members of the Kv1 family, Kv1.5, and Kv1.1 [9]. While carrying out this work, we made the serendipitous observation that cells bathed in extracellular reducing agents formed tighter seals more readily and lasted longer compared to ambient conditions. In the current report, we sought to quantitatively assess how reducing agents might contribute to high-quality recordings for experimenters of varying degrees of experience. We observed that reducing agents enabled higher resistance seals after break-in and promoted seal longevity regardless of whether a beginner, intermediate, or advanced patch clamper performed experiments with heterologous or primary cells (Figures 2–5).

The exact mechanism for the formation of a giga-ohm seal between the cell membrane and patch pipette has not been firmly established but consists of a strong interaction between the membrane lipids and the glass surface of the pipette. Forces between the cell membrane and pipette (electrostatic, van der Waals, hydration, double-layer, entropic origin) are both attractive and repulsive by nature [14,15]. There are several reports discussing the physical properties of an interaction between membrane and pipette through modeling [14,16,17], however the biological nature of the interaction taking into consideration the complexity of the membrane is not understood. Membrane, membrane-bound and intramembrane components including lipids, cholesterol, proteins and proteoglycans, lipid raft microdomains, and the cytoskeleton might all influence the contact between a cell with other surfaces [14,15]. For instance, membrane proteins such as the acetylcholine receptor (AChRs) that extend well above (>5 nm) the lipid bilayer and interact with other membrane-bound surfaces have been suggested to impede the interaction between the bilayer and glass pipette [18,19]. Lipids and lipid-rafts, cholesterol, and the intracellular cytoskeleton, alter membrane fluidity and create undulations in the membrane that may affect the contact between the patch pipette and the membrane [20,21]. Although we have not determined the exact mechanism for the increased seal formation in the presence of reducing agents, we speculate that it may involve alteration of key membrane or membrane-bound constituents (particularly in the extracellular environment), facilitating a more direct interaction between the membrane and the glass pipette. Evidence for this hypothesis is several fold: 1) Reducing agents (DTT and TCEP) break disulfide bonds between glycoproteins, proteoglycans, and the glycocalyx that form the structural environment above the lipid bilayer [22,23]. 2) DTT has been shown to acutely (<1 hr) lead to remodeling of the lipidome and lipid raft microdomains in cultured cells [24]. 3) Oxidizing agents (H_2_O_2_) appear to have the opposite effect on seal formation and longevity. 4) Finally, although we have not characterized this in detail, we find that application of reducing agents in the pipette solution during recording do not lead to any significant improvement in seal formation or longevity ruling out any instantaneous effect between the pipette and the membrane (data not shown).

Aside from the ionic composition of solutions and glass pipettes, there is limited information about external factors that help with giga-seals during patch clamping. It has been shown that the force exerted by constant laminar flow markedly prolongs the longevity of a seal [25]. In our study, we demonstrate that reducing agents in the bath solution greatly improve the formation and longevity of giga-ohm seals. In general, we have found that inclusion of a reducing agent in our recordings (when compatible with experimental objectives) can be very helpful with success of patch clamp experiments. This may be helpful in a variety of contexts. For example, it may help accelerate the learning of a beginner patch clamper, by helping them achieve seals more readily. Alternatively, for an experienced patch clamper working on challenging protocols that sample extreme voltages, the use of this manipulation may help complete otherwise impossible experiments. As a caveat, before considering using reducing agents to assist with the patch clamp technique, it should be understood how reducing conditions will impact the biological system (for example, this would not be an appropriate tool to use if the ion channel being studied exhibits intrinsic redox sensitivity). Where appropriate, the enhancement of seals with extracellularly applied reducing agents can potentially be used to increase the quality of recordings and reduce the amount of time required to collect valuable data. We encourage fellow patch clampers to give it a try, and hope this will be helpful when suitably applied.

## Data Availability

The data that support the findings of this study are available from the corresponding author, S.M.L., upon request.

## References

[1] Hamill OP, Marty A, Neher E, et al. Improved patch-clamp techniques for high-resolution current recording from cells and cell-free membrane patches. Pflugers Arch. 1981;391:85–10. doi: 10.1007/BF00656997 6270629

[2] Neher E, Sakmann B. Single-channel currents recorded from membrane of denervated frog muscle fibres. Nature. 1976;260:799–802. doi: 10.1038/260799a0 1083489

[3] Sakmann B, Neher E, eds. Single-channel recording. Boston (MA): Springer US; 1995.

[4] Hill CL, Stephens GJ. An introduction to patch clamp recording. Methods Mol Biol. 2021;2188:1–19. doi: 10.1007/978-1-0716-0818-0_1 33119844

[5] Rae JL, Levis RA. Glass technology for patch clamp electrodes. Methods Enzymol. 1992;207:66–92. doi: 10.1016/0076-6879(92)07005-9 1528127

[6] Priel A, Gil Z, Moy VT, et al. Ionic requirements for membrane-glass adhesion and giga seal formation in patch-clamp recording. Biophys J. 2007;92:3893–3900. doi: 10.1529/biophysj.106.099119 17369408 PMC1868979

[7] Maathuis FJM, Taylor AR, Assmann SM, et al. Seal-promoting solutions and pipette perfusion for patch clamping plant cells. Plant J. 1997;11(4):891–896. doi: 10.1046/j.1365-313X.1997.11040891.x 9161044

[8] Maguire AD, Plemel JR, Kerr BJ. Enrichment of adult mouse dorsal root ganglia neuron cultures by immunopanning. J Vis Exp. 2023. doi: 10.3791/64603 36912525

[9] Baronas VA, Yang RY, Kurata HT. Extracellular redox sensitivity of Kv1.2 potassium channels. Sci Rep. 2017;7:9142. doi: 10.1038/s41598-017-08718-z 28831076 PMC5567313

[10] Maki BA, Cummings KA, Paganelli MA, et al. One-channel cell-attached patch-clamp recording. J Vis Exp. 2014. doi: 10.3791/51629 PMC4188217 24961614

[11] Perkins KL. Cell-attached voltage-clamp and current-clamp recording and stimulation techniques in brain slices. J Neurosci Methods. 2006;154:1–18. doi: 10.1016/j.jneumeth.2006.02.010 16554092 PMC2373773

[12] Yan L, Fang Q, Zhang X, et al. Optimal pipette resistance, seal resistance, and zero-current membrane potential for loose patch or breakthrough whole-cell recording in vivo. Front Neural Circuits. 2020;14:34. doi: 10.3389/fncir.2020.00034 32714153 PMC7344171

[13] Roecker AJ, Layton ME, Pero JE, et al. Discovery of arylsulfonamide Nav1.7 inhibitors: IVIVC, MPO methods, and optimization of selectivity profile. ACS Med Chem Lett. 2021;12(6):1038–1049. doi: 10.1021/acsmedchemlett.1c00218 34141090 PMC8201757

[14] Suchyna TM, Markin VS, Sachs F. Biophysics and structure of the patch and the gigaseal. Biophys J. 2009;97:738–747. doi: 10.1016/j.bpj.2009.05.018 19651032 PMC2718145

[15] Corey DP, Stevens CF. Science and technology of patch-recording electrodes. In: Sakmann B Neher E, editors Single-channel recording. Boston (MA): Springer US; 1983. pp. 53–68.

[16] Ursell T, Agrawal A, Phillips R. Lipid bilayer mechanics in a pipette with glass-bilayer adhesion. Biophys J. 2011;101(8):1913–1920. doi: 10.1016/j.bpj.2011.08.057 22004745 PMC3192956

[17] Clarke RW. Theory of cell membrane interaction with glass. Phys Rev E. 2021;103:032401. doi: 10.1103/PhysRevE.103.032401 33862714

[18] Unwin N. Acetylcholine receptor channel imaged in the open state. Nature. 1995;373:37–43. doi: 10.1038/373037a0 7800037

[19] Baenziger JE, daCosta CJB. Molecular mechanisms of acetylcholine receptor–lipid interactions: from model membranes to human biology. Biophys Rev. 2012;5(1):1–9. doi: 10.1007/s12551-012-0078-7 28510176 PMC5425705

[20] Korade Z, Kenworthy AK. Lipid rafts, cholesterol, and the brain. Neuropharmacology. 2008;55:1265–1273. doi: 10.1016/j.neuropharm.2008.02.019 18402986 PMC2638588

[21] Bradley RP, Radhakrishnan R, Curvature–undulation coupling as a basis for curvature sensing and generation in bilayer membranes, Proceedings of the National Academy of Sciences. 2016;113:E5117–E5124. doi: 10.1073/pnas.1605259113. PMC5024647 27531962

[22] Alberch L, Yarema KJ. Chapter 3 - bioconjugation reactions in living cells: development, advances, and applications of glycan-specific technologies. In: Karp JM Zhao W, editors Micro- and nanoengineering of the cell surface. Oxford: William Andrew Publishing; 2014. pp. 43–62.

[23] Singh R, Whitesides GM. Reagents for rapid reduction of disulfide bonds in proteins. In: Crabb JW, editor Techniques in protein chemistry. Cambridge: Academic Press; 1995. pp. 259–266.

[24] Reinhard J, Mattes C, Väth K, et al. A quantitative analysis of cellular lipid compositions during acute proteotoxic ER stress reveals specificity in the production of asymmetric lipids. Front Cell Dev Biol. 2020;8:756. doi: 10.3389/fcell.2020.00756 32850859 PMC7417482

[25] Sinclair J, Olofsson J, Phil J, et al. Stabilization of high-resistance seals in patch-clamp recordings by laminar flow. Anal Chem. 2003;75(23):6718–6722. doi: 10.1021/ac0346611 16465721

